# Mechanism of Gene Regulation by a *Staphylococcus aureus* Toxin

**DOI:** 10.1128/mBio.01579-16

**Published:** 2016-10-25

**Authors:** Hwang-Soo Joo, Som S. Chatterjee, Amer E. Villaruz, Seth W. Dickey, Vee Y. Tan, Yan Chen, Daniel E. Sturdevant, Stacy M. Ricklefs, Michael Otto

**Affiliations:** a Pathogen Molecular Genetics Section, Laboratory of Bacteriology, National Institute of Allergy and Infectious Diseases, National Institutes of Health, Bethesda, Maryland, USA; b Genomics Unit, Research Technology Branch, Rocky Mountain Laboratories, National Institute of Allergy and Infectious Diseases, National Institutes of Health, Hamilton, Montana, USA

## Abstract

The virulence of many bacterial pathogens, including the important human pathogen *Staphylococcus aureus*, depends on the secretion of frequently large amounts of toxins. Toxin production involves the need for the bacteria to make physiological adjustments for energy conservation. While toxins are primarily targets of gene regulation, such changes may be accomplished by regulatory functions of the toxins themselves. However, mechanisms by which toxins regulate gene expression have remained poorly understood. We show here that the staphylococcal phenol-soluble modulin (PSM) toxins have gene regulatory functions that, in particular, include inducing expression of their own transport system by direct interference with a GntR-type repressor protein. This capacity was most pronounced in PSMs with low cytolytic capacity, demonstrating functional specification among closely related members of that toxin family during evolution. Our study presents a molecular mechanism of gene regulation by a bacterial toxin that adapts bacterial physiology to enhanced toxin production.

## IMPORTANCE

Toxins play a major role in many bacterial diseases. When toxins are produced during infection, the bacteria need to balance this energy-consuming task with other physiological processes. However, it has remained poorly understood how toxins can impact gene expression to trigger such adaptations. We found that specific members of a toxin family in the major human pathogen *Staphylococcus aureus* have evolved for gene regulatory purposes. These specific toxins interact with a DNA-binding regulator protein to enable production of the toxin export machinery and ascertain that the machinery is not expressed when toxins are not made and it is not needed. Our study gives mechanistic insight into how toxins may directly adjust bacterial physiology to times of toxin production during infection.

## INTRODUCTION

*Staphylococcus aureus* is an important bacterial pathogen that imposes a significant global health care burden (1). This bacterium can cause a variety of diseases, ranging from moderately severe skin and soft tissue infections to more serious and frequently fatal infections, such as osteomyelitis, pneumonia, endocarditis, and sepsis. Antibiotic resistance, such as in methicillin-resistant *S. aureus* (MRSA), is widespread among *S. aureus* isolates and significantly impacts treatment and infection outcome (2). While MRSA has traditionally predominantly presented a problem for hospitalized and predisposed patients, it has more recently also emerged as a considerable cause of community-associated infections affecting healthy individuals (3).

In addition to antibiotic resistance, the success of *S. aureus* as a pathogen is attributed to its vast array of virulence genes. These include factors that facilitate adhesion to host cells, nutrient acquisition by tissue degradation, and evasion of host defenses. Immune evasion is, to a large extent, due to cytolytic toxins with the capacity to kill immune cells, among which the most important are α-toxin, the bicomponent leukotoxin family, and the phenol-soluble modulin (PSM) peptides (4).

In *S. aureus*, PSMs constitute a group of seven different peptides that are encoded by three different loci in the bacterial genome (5, 6). PSM peptides can be grouped into α- and β-type PSMs. The α-type peptides are ~20 to 25 amino acids in size and, in *S. aureus*, comprise PSMα1-4 and the δ-toxin, which are encoded by the *psm*α and *hld* loci, respectively. *S. aureus* PSMβ1 and PSMβ2 are members of the larger (~45-amino-acid) β-type PSMs and are encoded by the *psm*β locus. The *hld* gene is embedded within RNAIII, the intracellular effector of the accessory gene regulator (*agr*) system (7). In *S. aureus*, PSMα peptides and δ-toxin are highly abundant, whereas only minute amounts of PSMβ peptides are present under common *in vitro* conditions (8). PSM peptides form amphipathic α-helical structures capable of forming pores in artificial membranes at micromolar concentrations (9). Several members of the PSM family, in particular, PSMα peptides, have been shown to cause lysis of a wide variety of cells, including human neutrophils, erythrocytes, and osteoblasts (6, 10, 11). Recent studies have underlined the importance of PSMs as mediators of a number of staphylococcal diseases, including skin and soft tissue infections, sepsis, osteomyelitis, and atopic dermatitis (6, 12–14).

Toxin production requires the cell to make physiological adjustments, in particular in the case of PSMs, which are produced at extremely high levels, reaching ~60% of the total secreted protein mass in *S. aureus* (15). This task may be accomplished by putting toxin genes and other genes that are required in times of toxin production under common regulation. Probably for that reason, virtually all *S. aureus* toxins are under the control of the accessory gene regulator (*agr*) quorum-sensing system, which is also in charge of a wide variety of metabolic adaptations (16). Possibly owing to their high-level production, PSM synthesis is exceptionally strictly regulated by direct binding of the AgrA response regulator to *psm* operon promoters (17), resulting in complete absence of PSMs in mutants with a dysfunctional *agr* system (6).

Theoretically, another way of adjusting gene expression to toxin production is by direct gene regulatory functions of the toxins themselves. However, reports on gene regulatory functions of toxins in *S. aureus* or other bacterial pathogens are rare. Pleiotropic effects of *S. aureus* toxins have been reported occasionally (18, 19), but it has remained unclear whether they were due to a genuine gene regulatory mechanism of the toxin. This is because only the regulatory outcome but not the underlying mechanism was addressed. Furthermore, PSMα peptides have been convincingly shown to affect the expression of α-toxin, albeit only during very specific times during growth (20); however, also in that case, the underlying mechanism has remained undefined. Of note, in one case, the observed regulatory effects were later found to be probably due to unintended mutations in the *agr* global regulatory system (21), which occur frequently in *S. aureus* (22) and without proper genetic complementation may wrongly indicate a gene regulatory function of the locus under investigation.

We recently reported the discovery of the PSM-exporting system Pmt (15). Pmt is an ATP-binding cassette (ABC) transporter encoded by four genes, *pmtA* to -*D* (*pmtA*-*D*). Pmt is essential, as in its absence, PSMs accumulate inside the bacterial cytosol, which is lethal to the bacteria. Pmt is ubiquitously present among all *Staphylococcus* species and is capable of exporting all types of PSM peptides. Upstream of the *pmtA*-*D* genes is a gene predicted to encode a GntR-type transcriptional regulator. In the present study, we show that the expression of *pmt* is under the repression of this regulator protein, which we named PmtR. Binding of PmtR to the operator site of the *pmt* promoter causes repression of the *pmt* cluster. PSMs bind to PmtR and disrupt the PmtR-*pmt* promoter complex, which enables *pmt* transcription. Thus, PSMs positively influence the expression of *pmt* to facilitate their own export. Our findings provide previously unavailable molecular evidence for a regulatory mechanism of a staphylococcal toxin.

## RESULTS

### PSMα peptides positively regulate the expression of *pmt*.

**FIG 1:**
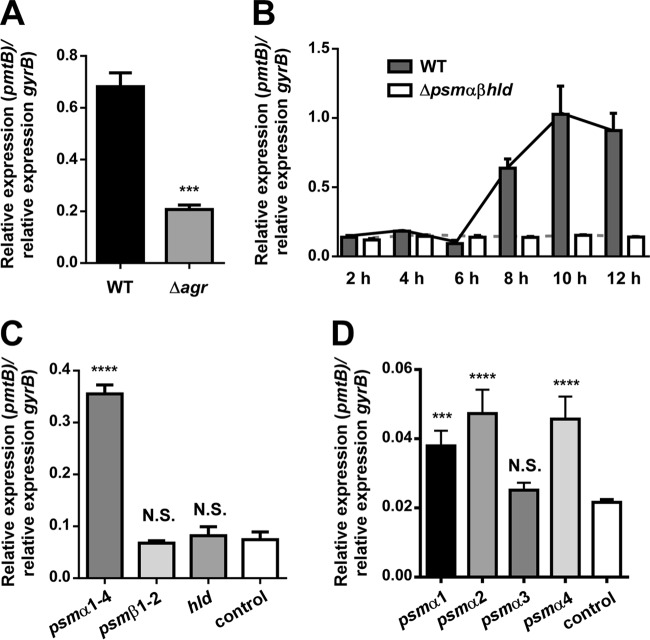
PSMs trigger expression of *pmt*. (A) Expression of *pm*t in wild-type (WT, strain MW2) and isogenic Δ*agr* mutant strains. A sequence within the *pmtB* gene was used in all qRT-PCR experiments to measure *pmt* transcription. ***, *P* < 0.001 (unpaired *t* test). (B) Growth phase-dependent expression of *pmt* in WT and isogenic Δ*psm*αβ*hld* mutant strains. (C) Expression of *pmt* in strains expressing the *psm*α or *psm*β operon or the *hld* gene in a PSM-negative (Δ*agr*) background. (D) Expression of *pm*t in strains expressing single *psm*α genes in a PSM-negative (Δ*psm*αβ*hld*) background. (C, D) Xylose at 0.5% was used to trigger expression of PSMs in the pKX (C) or pTX (D) plasmid background. Controls harbor the respective control plasmids. ***, *P* < 0.001; ****, *P* < 0.0001; N.S., not significant (one-way ANOVA with Dunnett’s posttest versus the control). All error bars represent SDs.

To analyze whether PSMs are involved in the regulation of *pmt* genes, we used quantitative real-time PCR (qRT-PCR) to compare the expression of *pmt* in a wild-type *S. aureus* strain (MW2) with that in (i) an isogenic PSM regulator *agr* mutant and (ii) an isogenic mutant in which all of the *psm* genes were deleted or their expression was abolished (Δ*psm*αβ*hld*). While *pmt* expression was strongly growth phase dependent, in accordance with regulation by the *agr* quorum-sensing system, it was significantly reduced in the Δ*agr* and Δ*psm*αβ*hld* mutants, indicating that PSMs positively regulate the expression of *pmt* genes (Fig. 1A and B).

Then, to investigate which PSM subtypes are involved in *pmt* regulation, we expressed the *psm*α1-4, *psm*β1-2, or *hld* gene from plasmids in a PSM-negative background and measured *pmt* expression. These experiments revealed that the *psm*α locus is predominantly involved in *pmt* regulation, as only the *psm*α1-4 expression plasmid, but not the *psm*β1-2 or *hld* expression plasmid, led to significant changes in *pmt* expression (Fig. 1C).

To investigate whether all or only specific PSMα peptides regulate *pmt*, we expressed all single *psm*α genes in the PSM-negative background strain. Only plasmids expressing PSMα1, PSMα2, or PSMα4, but not a plasmid expressing PSMα3, led to significant increases in *pmt* expression (Fig. 1D). Together, these results indicated that PSMα1, PSMα2, and PSMα4 peptides are involved in the regulation of *pmt*.

### PmtR negatively regulates *pmt* expression.

**FIG 2:**
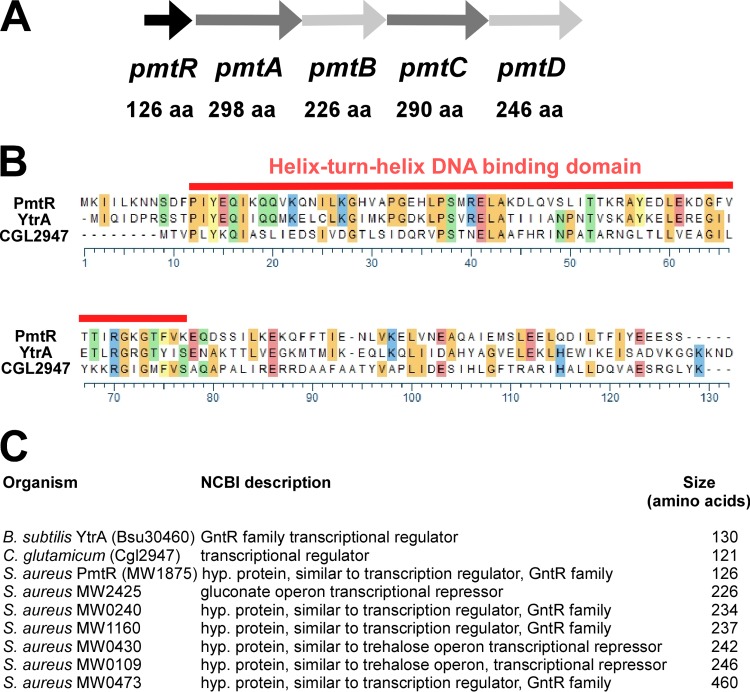
The PmtR-encoding gene and protein. (A) Location of the *pmtR* gene in the *pmt* locus. aa, amino acids (length of the encoded protein product). (B) ClustalW alignment of PmtR with two other YtrA subfamily GntR-type transcriptional regulators, YtrA of *B. subtilis* (39% homology) and Cgl2947 of *C. glutamicum* (27% homology). (C) GntR-type regulators of *S. aureus*. Sizes are based on the MW2 genome information. Note that PmtR is the only small-size, YtrA-type, GntR-type regulator in *S. aureus*.

Immediately upstream of the *pmt* gene cluster, the *S. aureus* genome contains a conserved gene encoding a putative transcriptional regulator (Fig. 2A). We named that gene *pmtR*, as we show in the following that it is involved in regulation of the *pmt* gene locus. Similarity to the *Bacillus subtilis* YtrA (39% homology) and *Corynebacterium glutamicum* Cgl2947 (27% homology) proteins characterizes the PmtR protein as a member of the YtrA subfamily of GntR-type transcriptional regulators (23, 24) (Fig. 2B). There are seven GntR-type regulators in the *S. aureus* genome. PmtR is the only protein of *S. aureus* belonging to the YtrA subfamily, which is characterized by a very small size, containing only one α-helical region in addition to the N-terminal DNA-binding helix-turn-helix (HTH) motif (24) (Fig. 2C).

**FIG 3:**
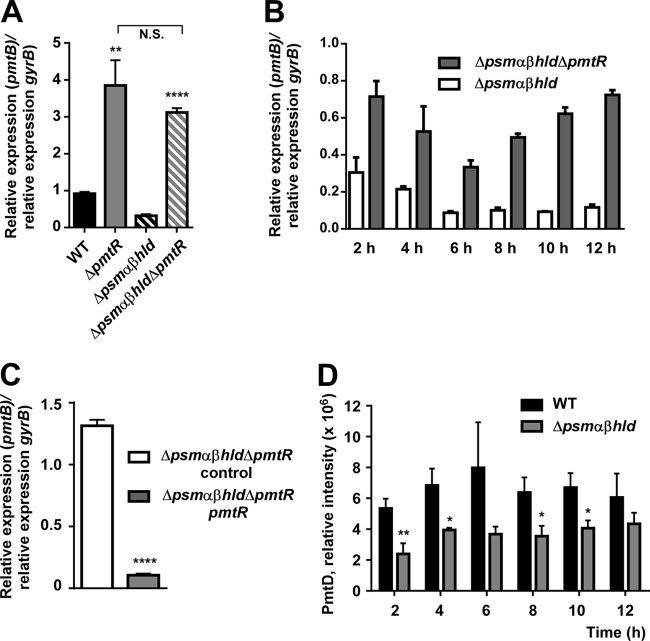
PmtR controls *pmt* expression. (A) Comparison of *pmt* expression in wild-type (WT) and isogenic deletion strains. **, *P* < 0.01 (versus WT); ****, *P* < 0.0001; N.S., not significant (one-way ANOVA with Tukey’s posttest versus Δ*psm*αβ*hld* mutant). (B) Growth-dependent expression of *pmt* in Δ*psm*αβ*hld* and isogenic Δ*pmtR* deletion strains. Differences between the Δ*psm*αβ*hld* and Δ*psm*αβ*hld* Δ*pmtR* mutant strains are significant at every time point (*P* < 0.05 [unpaired *t* tests]). (C) Genetic complementation of Δ*psm*αβ*hld* Δ*pmtR* mutant with plasmid-carried *pmtR* under constitutive expression. Control, with corresponding control plasmid. ****, *P* < 0.0001 (unpaired *t* test). (D) Immunoblot analysis of PmtD expression over growth. Shown are values from three samples at each time point obtained by densitometry of PmtD bands. *, *P* < 0.05; **, *P* < 0.01 (unpaired *t* test). All error bars represent SDs.

To confirm that PmtR regulates *pmt* expression, we first created isogenic *pmtR* deletions in the MW2 wild-type and PSM-negative isogenic Δ*psm*αβ*hld* mutant strains. Expression of *pmt* was significantly increased in both deletion strains compared to the respective parent strains, and there was no significant difference in *pmt* expression between the two *pmtR* deletion strains (Fig. 3A). Furthermore, absence of PSMs abrogated the growth phase dependence of *pmt* expression (Fig. 3B). When *pmtR* was provided in *trans* to the Δ*psm*αβ*hld* Δ*pmtR* strain, a significant decrease in *pmt* expression was observed (Fig. 3C). Western blot analysis with specific MAbs developed against the PmtD transmembrane protein part of Pmt confirmed the regulation of Pmt by PmtR on the protein level (Fig. 3D). These results confirmed that *pmtR* negatively regulates *pmt* expression. Moreover, they indicated that there is no other *pmtR*-independent mode of *pmt* regulation by PSMs.

### PmtR specifically regulates *pmt*.

To assess if PmtR regulates any other genes in addition to the *pmt* cluster, we compared the genome-wide gene expression profiles of the Δ*psm*αβ*hld* and Δ*psm*αβ*hld* Δ*pmtR* strains by microarray analysis. The microarray results showed that all of the *pmt* genes, and only those, showed strong changes in expression, dependent on the presence of *pmtR* (Table 1). There was only one additional gene that showed a small yet significant *pmtR*-dependent change in expression. This gene is the next gene downstream of the *pmt* locus that is transcribed in the same direction as the *pmt* genes, suggesting that the change in its expression may be explained by limited transcriptional readthrough. These results demonstrated that the *pmt* cluster is the only regulatory target of PmtR and also confirmed that the *pmtA*-*D* genes form a regulatory unit.

**TABLE 1:** Microarray comparison of gene expression in Δαβ*hld* and Δαβ*hld* Δ*pmtR* mutant strains^a^

New NCBI ID	Old ID	Gene	Function	*P* value	Fold change^b^
MW_RS10215	MW1875	*pmtR*	GntR type transcriptional regulator	0.01817	−50.9
MW_RS10210	MW1874	*pmtA*	ABC transporter ATPase domain	0.01905	7.3
MW_RS10205	MW1873	*pmtB*	ABC transporter membrane domain	0.01921	14.6
MW_RS10200	MW1872	*pmtC*	ABC transporter ATPase domain	0.01921	8.0
MW_RS10195	MW1871	*pmtD*	ABC transporter membrane domain	0.01921	11.7
MW_RS10185	MW1869	Hypothetical	Unknown	0.04342	3.0

a All of the differentially regulated genes that passed significance tests are shown.

b Δ*pmtR* mutant versus control.

### PmtR binds to the *pmt* promoter region.

**FIG 4:**
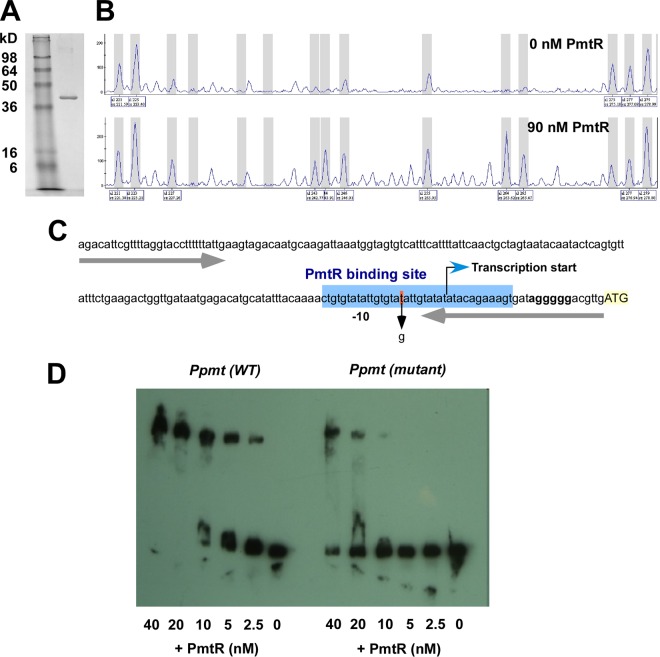
PmtR binds to a specific site in the *pmtR* promoter region. (A) Purified PmtR-GST fusion protein. (B) Footprinting analysis showing PmtR-protected region. (C) PmtR binding site as determined by footprinting analysis. The ribosomal binding site (Shine-Dalgarno sequence) is in bold; the ATG start codon of the *pmtR* gene is highlighted in yellow. The transcription start site was determined by 5′ RACE. The site of the 1-bp mutation used for panel D is red. (D) EMSA of PmtR binding to labeled wild-type and mutated (see panel C) Ppmt* promoter fragment.

Transcriptional repressors inhibit the expression of target genes by binding to a DNA operator site in a sequence-specific manner, blocking interaction of RNA polymerase with the promoter. The overlapping arrangement and common orientation of the *pmtR* and *pmtA*-*D* genes suggest that they have one shared promoter upstream of *pmtR* that controls the transcription of the entire *pmtR*/*pmtA*-*D* operon, a notion supported also by our microarray results. In order to determine the PmtR binding site, we amplified the putative promoter region of *pmt* (P*pmt*) and purified PmtR as a glutathione *S*-transferase (GST) fusion protein (Fig. 4A). We then incubated purified GST-PmtR with P*pmt* and performed a DNase protection assay, followed by DNA fragment analysis (Fig. 4B). This experiment demonstrated that PmtR binds to the *pmt* promoter region and identified a 36-bp sequence overlapping the determined transcription start site as the PmtR binding site (Fig. 4C). To further confirm the involvement of this region in PmtR binding and demonstrate the specificity of the interaction, we introduced a 1-bp mutation in the 36-bp sequence (Fig. 4C), which, as shown by an electrophoretic mobility shift assay (EMSA), led to a strong decrease (~10 times) in binding (Fig. 4D). Moreover, PmtR did not bind to a random DNA sequence (see Fig. S1 in the supplemental material). These findings thus clearly showed specific binding of PmtR to the *pmt* promoter.

### Specific PSMs bind to PmtR, disrupt the PmtR-P*pmt* complex, and lead to *pmt* transcription.

**FIG 5:**
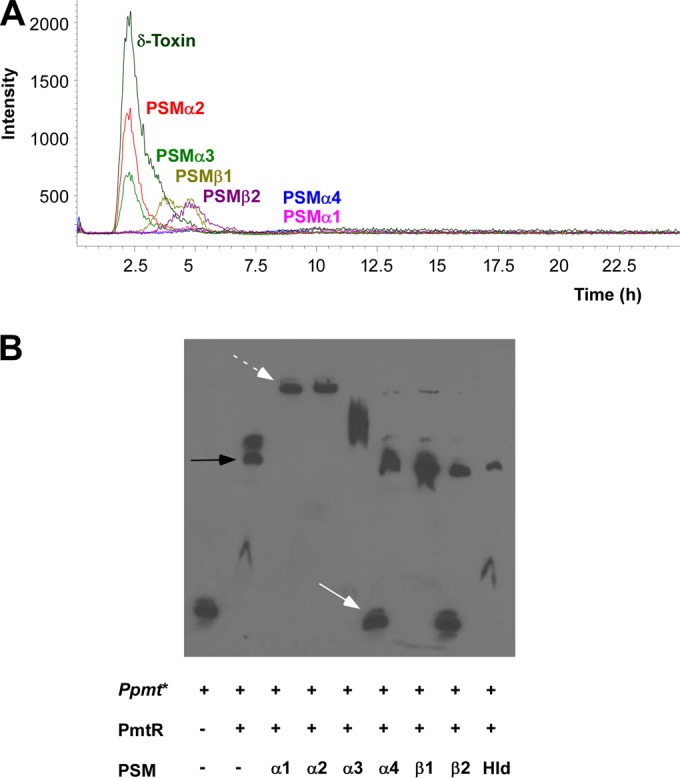
Specific PSMs bind to PmtR and interact with the PmtR-*pmt* promoter complex. (A) HPLC-MS analysis of binding of specific PSMs to a PmtR affinity column. (B) EMSA of the labeled Ppmt* promoter fragment with PmtR and different PSMs. Black arrow, Ppmt*/PmtR complex. White arrow, released free Ppmt*. White dashed arrow, Ppmt*-PSM complex. Note that the bands marked by dashed white arrows do not comprise PmtR, as shown by specific antibodies in native SDS-PAGE and Western blotting (see Fig. S2 in the supplemental material).

To analyze whether the impact of PSMs on *pmt* transcription is due to binding to PmtR and subsequent release of the PSM-PmtR repressor complex from P*pmt*, we first analyzed the binding of PSMs to PmtR by a liquid chromatography-mass spectrometry (MS) approach. This had become necessary as other methods (such as native polyacrylamide gel electrophoresis) to demonstrate protein-protein interaction failed because both PSMs and PmtR aggregate at the quite high concentrations of protein those other methods require. We used a glutathione column to which the GST-PmtR fusion protein was bound by GST-glutathione interaction and then passed different PSMs over the column. The δ-toxin, PSMα2, and PSMα3 showed no interaction with PmtR. PSMβ1 and PSMβ2 showed slight retention. The results for PSMα2 are inconsistent with those achieved by genetic evidence (Fig. 1D), which may be due to the different PSM and PmtR concentrations present *in vivo*. Notably, PSMα1 and PSMα4 were completely retained, demonstrating strong interaction with PmtR (Fig. 5A).

To explore whether PSMs directly interfere with the PmtR-P*pmt* complex and release PmtR from P*pmt*, we then performed EMSAs with biotin-labeled P*pmt* (Ppmt*), purified PmtR fusion protein, and synthetic PSM peptides. The Ppmt* PCR fragment migrated slowly upon addition of the purified PmtR protein, indicating complex formation between PmtR and Ppmt* (Fig. 5B), which was further confirmed by reaction of that band with anti-PmtR antiserum (see Fig. S2 in the supplemental material). Administration of PSMα4 or PSMβ2 led to free Ppmt* DNA, while administration of PSMα1 and PSMα2 produced a pronounced slowly migrating band that, according to Western blot analysis (see Fig. S2), was devoid of PmtR. It thus likely represents Ppmt* DNA with attached PSMs. In fact, by using a random DNA fragment, we found that several PSMs, in particular, PSMα1 and PSMα2, have some propensity to attach to DNA in a nonspecific manner (see Fig. S1). These findings are to be interpreted in a way suggesting that some PSMs, such as PSMα1 and PSMα2, nonspecifically bind to DNA and thus remain bound to the Ppmt* DNA fragment in the EMSA after PmtR release. The δ-toxin and PSMα3 showed no bands indicating binding to or displacement of PmtR (Fig. 5B). PSMβ1 only showed a very faint complex band with DNA, similar to those observed with PSMα1 and PSMα2. These results indicate that PmtR is displaced from the *pmt* promoter by PSMα1, PSMα2, PSMα4, or PSMβ2 but not to a significant degree by the δ-toxin, PSMα3, or PSMβ1. Overall, these results are in good agreement with the qRT-PCR and GST-PmtR interaction results. Only the results for PSMα2, which showed a regulatory effect by genetic analysis and displaced PmtR from the DNA in the EMSA but lacked retention in the GST-PmtR interaction test, were not entirely consistent. The fact that the observed PmtR binding and displacement activities of synthetic PSMβ2 (Fig. 5) did not translate to a measurable impact of the *psm*β operon on *pmt* expression (Fig. 1C) is likely due to the comparably minimal expression of the *psm*β operon and PSMβ peptides in *S. aureus*, even when expressed from a plasmid (6, 8). Together, our results identify, in particular, PSMα4 as the main PSM that interacts with PmtR to facilitate derepression and transcription of the *pmt* operon. This is especially noteworthy given that PSMα4, in contrast to the other PSMα peptides, has very low cytolytic activity (6), demonstrating functional specification among the peptides encoded by the *psm*α operon.

### Restriction of Pmt expression by PmtR is important for bacterial growth. 

The biological purpose of regulating toxin export is to limit wasteful production of the transport machinery when it is not needed. Deletion of the PmtR repressor is not expected to result in increased PSM secretion compared to that of the wild-type strain, as the presence of PSMs already ensures sufficient production of Pmt for PSM secretion in a wild-type strain via PmtR. Accordingly, PSM concentrations were unchanged in wild-type versus Δ*pmtR* mutant strains (see Fig. S3 in the supplemental material). However, deletion of *pmtR* led to a slight but significant growth defect in synthetic medium, in accordance with the idea that futile production of Pmt during growth stages without PSM production is wasteful for cells (see Fig. S3).

### Other targets of PSM gene regulation.

We hypothesized that the derepression mechanism by which PSMs regulate gene expression and which we describe here may potentially be used by *S. aureus* for more than regulation of only the *pmt* transporter operon. Previously, we found no targets of PSM-dependent gene regulation in *S. aureus* when using single *psm* mutants (17). We then had concluded that PSMs do not have gene regulatory effects, a notion that we had to correct given the present findings. Considering that effects may only become apparent when no PSMs are present at all, because PSMs may complement each other in their regulatory effects, we compared gene expression in the Δ*psm*αβ*hld* complete PSM deletion mutant with that in the corresponding wild-type strain by microarray analysis (see Table S1 in the supplemental material). We did indeed detect gene regulatory effects of PSMs that had not become apparent previously with single mutants. Among genes positively regulated by PSMs, in addition to the *pmt* and *pmtR* genes, were several protease genes, and among those negatively regulated by PSMs were predominantly genes belonging to the urease and capsular polysaccharide synthesis operons. Furthermore, the general secretion protein gene *secY* and an adjacent gene were negatively regulated, suggesting that to cope with increased secretion of PSMs, general protein secretion is downregulated. Interestingly, the α-toxin gene *hla* was not among the PSM-regulated targets under the conditions used in our assay, emphasizing the previously reported pronounced growth phase dependence of that regulatory effect of PSMs (20). We did not find GntR-type regulator-encoding genes next to any of the PSM-regulated genes located in a way similar to the arrangement of the *pmtR*/*pmt* operon, indicating that the regulatory impact of PSMs on those genes may occur via different regulators or mechanisms, in a potentially indirect fashion, or that the regulators responsible for PSM-dependent gene regulation in those cases are not located next to the regulated genes.

## DISCUSSION

Limiting the expression of toxins to situations in which they are needed is critical for bacterial energy conservation. Regulation of PSM expression and secretion is a particularly challenging task for staphylococci. This is because PSMs not only have multiple key roles in the infectious and commensal lifestyles of staphylococci and are often secreted in huge amounts (5, 15, 25) but also become deleterious for bacterial survival if obstruction of export leads to their accumulation in the cytosol (15).

**FIG 6:**
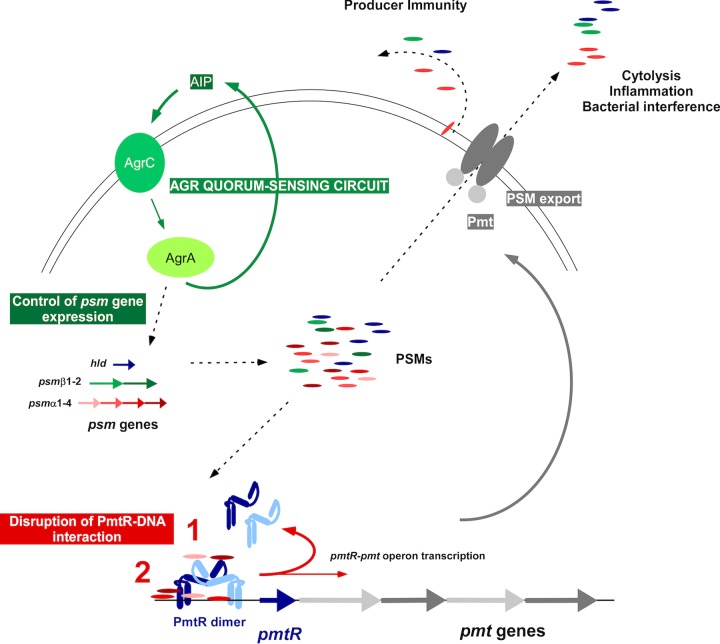
Scheme of PSM export, regulation, and interaction with *pmt*. The different *psm* genes are shown at the left. Their expression is under the control of the *agr* quorum-sensing system, which occurs by direct binding of AgrA to *psm* promoters. Accumulated intracellular PSMs (in particular, PSMα4) disrupt the *pmt* promoter-PmtR repressor complex, leading to transcription of the *pmtR* and *pmtABCD* genes. The resulting production of the Pmt PSM exporter enables PSM secretion, with secreted PSMs exhibiting their characteristic impact on cytolysis, inflammation, and possibly bacterial interference. Pmt also protects the producer cell from PSM cytotoxicity. Note two potential hypotheses for how PSMs may disrupt the *psm* promoter-PmtR repressor complex: (i) binding to the C-terminal PmtR effector-binding domain (canonical model) and (ii) direct disruption of DNA-PmtR interaction. AIP, autoinducing peptide.

One common way for bacteria to balance toxin production with other physiological needs is by putting the expression of toxins under the control the same global regulator, such as *agr* in staphylococci (16, 26). Another is for toxins to directly regulate the expression of other genes. While such gene regulatory functions of *S. aureus* toxins have been described previously (19, 20), it is poorly understood how they work on a mechanistic level. We show here that PSMs regulate their own export by interacting with a DNA-binding repressor, PmtR, facilitating transcription of the *pmt* genes (Fig. 6). The PSM/PmtR-controlled transcriptional unit comprises the repressor gene itself, likely to ensure fine triggering of the regulatory circuit by balancing repressor with effector concentrations. Thus, direct control of Pmt expression by PSMs in the cytosol ensures timely connection of PSM production with PSM export and avoids fatal intracellular PSM accumulation and futile production of the secretion machinery.

PmtR is a GntR-type regulator of the YtrA subfamily and the only representative of that subfamily in *S. aureus*. The X-ray crystal structure of YtrA-type Cgl2947 of *C. glutamicum* has recently been solved, which revealed a characteristic HTH DNA-binding domain at the N terminus and a fishhook-like C-terminal domain (23). Like all GntR-type regulators, Cgl2947 binds DNA as a homodimer. It has been suggested that effectors disrupt homodimer formation by binding to the C-terminal fishhook (23). However, others have noted that this domain is too small in the YtrA-type regulators for efficient effector binding (27). Our results indicate a possible alternative model of effector interaction with YtrA-type regulators, inasmuch as they suggest PmtR-independent binding of the PSM effectors to the DNA (see Fig. S1). It is thus possible that PSMs displace the PmtR dimer from the operator site by directly affecting the site of DNA-PmtR interaction (Fig. 6). DNA site specificity of this mechanism would be based on the specific interaction with PmtR that we show in our study. This potential novel mechanism of effector involvement with GntR-type regulators needs to be further explored in detail.

Recent studies have revealed pronounced functional specification among PSMs. For example, only some PSMs are cytolytic (6, 8) and only the δ-toxin degranulates mast cells (13). Our findings indicate functional specification also regarding the gene regulatory function of PSMs, inasmuch as only PSMα1, PSMα2, and PSMα4 were able to regulate *pmt* transcription. Low-level cytolytic PSMα4, in particular, consistently emerged in all assays as PmtR binding, disrupting the P*pmt*-PmtR complex, and facilitating transcription, while results for other PSMs, such as PSMα2, were not entirely consistent in the genetic and biochemical assays. In contrast, strongly cytolytic PSMα3 did not exhibit a regulatory function, suggesting that structural features underlying interaction with the PmtR-DNA complex are different from those that promote cytolysis and are possibly, to a certain extent, mutually exclusive. Interestingly, these findings indicate that the peptides encoded in the *psm*α operon, which most likely arose by gene duplication, have adopted different functions during evolution. Last, while not of biological impact in *S. aureus* because of low production, the capacity of a member of the PSM β-type to bind to PmtR and displace it from the *pmt* promoter indicates that PSMβ peptides may also have gene regulatory functions, in particular when strongly produced, such as in *S. epidermidis* (8).

In conclusion, our study shows how PSMs avoid fatal cytosolic accumulation by a direct gene regulatory mechanism controlling secretion and how this mechanism avoids unnecessary production of the PSM export machinery when PSMs are not produced. It presents mechanistic evidence for a gene regulatory function exerted by a staphylococcal toxin family and highlights the multiplicity of functions that PSMs have in staphylococcal physiology.

## MATERIALS AND METHODS

### Bacterial strains, plasmids, culture conditions, and PSM quantification.

The bacterial strains, plasmids, and primers used in this study are described in Table S2 in the supplemental material. *Escherichia coli* and *S. aureus* strains were grown in tryptic soy broth (TSB), with antibiotic selection as required, at 37°C with shaking at 180 rpm. Xylose (0.5%) was added whenever stated to induce gene expression from plasmids. PSMs were quantified in bacterial culture filtrates by reversed-phase high-performance liquid chromatography (RP-HPLC)–electrospray ionization–MS (RP-HPLC/ESI-MS) (28).

### Construction of a deletion mutant and gene expression plasmids.

Deletion of the *pmtR* gene was achieved by a pKOR1-based allelic replacement procedure as previously described (29). Briefly, ~1-kb fragments up- and downstream of the *pmtR* gene were PCR amplified with MW2 genomic DNA as the template and primers pmtR-P1 and pmtR-P2 and primers pmtR-P3 and pmtR-P4, respectively. The PCR fragments were fused by overlap PCR and subsequently cloned into vector pKOR1. The resulting vector was transformed into the recipient strains by electroporation, and the standard allelic replacement procedure was performed as previously described (29).

The plasmid for *pmtR* gene expression was created by amplifying the *pmtR* gene from MW2 genomic DNA with primers pmtR-for and pmtR-rev. The PCR product was cloned into constitutive expression vector pTX_Δ_ (6). For construction of pKX PSM expression constructs, pTX plasmids containing *psm*α1-4, *psm*β1-2, or *hld* genes (15) were cloned into pKX15 (30). The GST-PmtR fusion protein expression construct was created by amplifying the *pmtR* gene from strain MW2 with primers GST-pmtR-for and GST-pmtR-rev. The resulting PCR product was cloned into the vector pGEX-4T-1.

### RNA isolation, qRT-PCR, and microarray experiment.

RNA was isolated as previously described, with an RNeasy minikit (Qiagen) (31) from bacterial cultures grown to the stationary growth phase (10 h) unless otherwise indicated. The resulting RNA was treated with DNase I (Amersham Biosciences). cDNA was synthesized and labeled according to the manufacturer’s suggestions for Affymetrix antisense genome arrays as previously described (31). A gel shift assay with NeutrAvidin (Pierce Biotechnology) was performed to estimate the labeling efficiency based on the instructions from Affymetrix. Biotinylated *S. aureus* cDNA was hybridized to custom Affymetrix GeneChips (RMLChip 3) with 96% coverage of genes from MW2 (2,534 probe sets of 2,632 open reading frames) and scanned according to standard GeneChip protocols (Affymetrix). Affymetrix GeneChip Operating Software (GCOS v1.4; Affymetrix, Santa Clara, CA) was used to perform the preliminary analysis of the custom chips at the probe set level. Subsequent data analysis was performed as previously described (31). qRT-PCR was performed as previously described (6). cDNA was generated from RNA isolated from three independent experiments.

### Determination of transcription start site.

5ʹ RACE (rapid amplification of cDNA ends) was used to determine the transcription start site of the *pmt* operon (in front of the *pmtR* gene) and performed with the 5ʹ RACE System For Rapid Amplification of cDNA Ends kit (Invitrogen). Primer Pmtrace1 was used as the RNA adapter. PCR was performed with primers Pmtrace1 and Pmtrace2. PCR products were purified and then sequenced with Pmtraceseq. Mutations were confirmed by DNA sequencing.

### PmtD protein expression.

Expression of PmtD was measured with monoclonal antibodies (MAbs) that were developed by Precision Antibody, Columbia, MD, against a peptide from the PmtD sequence. Equal amounts of bacterial cells, as determined by measurement of optical density at 600 nm, were harvested by centrifugation from cultures grown in TSB at 37°C. The cells were resuspended in phosphate-buffered saline and lysed with glass beads. The lysates were separated on a 15% SDS-polyacrylamide gel and transferred to a nitrocellulose membrane. PmtD expression was detected by Western blotting with PmtD-specific IgG from hybridoma cell line 2D1 and quantified on a Typhoon Trio+ Variable Mode Imager.

### PmtR fusion protein purification.

A culture of *E. coli* BL21(DE3) harboring the *gst-pmtR* construct was grown overnight. The bacterial cells were collected by centrifugation, washed and resuspended in resuspension buffer (10 mM Tris, 150 mM NaCl, 1 mM EDTA, pH 8.0), and lysed with a French press, and the resulting lysate was centrifuged. The supernatant was mixed with glutathione-Sepharose 4B resin and shaken for 1 h at 4°C. The resin was then packed into a column and washed with resuspension buffer containing 0.1% Triton X-100, and the GST-PmtR fusion protein was eluted with elution buffer {10 mM reduced glutathione, 5 mM dithiothreitol (DTT), 25 mM Tris, 0.5 mM EDTA, 0.1% 3-[(3-cholamidopropyl)-dimethylammonio]-1-propanesulfonate (CHAPS), pH 8}. Finally, the protein was dialyzed in 50 mM Tris-Cl (pH 8)--50 mM EDTA--100 mM NaCl.

### DNase footprinting and fragment analysis.

For DNase footprinting analysis, the *pmt* promoter region was amplified from MW2 genomic DNA with primers PpmtR-for and PpmtR-rev and cloned into the pCR 2.1 TOPO cloning vector (Life Technologies). Sequence fidelity of the cloned PCR fragment was confirmed by DNA sequencing. Fluorescently labeled promoter regions were PCR amplified from the plasmids such that only one strand was labeled. The 5′ 6-carboxyfluorescein (FAM) fluorescently labeled promoter regions were amplified with a 5′ FAM-labeled primer (*M13L or *M13R) complemented with an unlabeled primer (M13R or M13L, respectively). Labeled PCR fragments were purified, quantified, and adjusted to 100 nM. Footprinting assays were performed as described by Yindeeyoungyeon and Schell (32), with some modifications. Briefly, binding reactions were set up in 10-µl volumes that contained 10 mM Tris-HCl (pH 7.5), 5 mM KCl, 1 mM EDTA, 2 mM DTT, 8% glycerol, 10 nM FAM-labeled DNA fragment, the indicated concentration of purified GST-PmtR, and bovine serum albumin to bring the total protein concentration to 4.5 mg/ml. After incubation at 30°C for 30 min, samples were equilibrated at 26°C and 5 μl of DNase I (3 × 10^−4^ U/µl) was added. Samples were incubated for 5 min, and digestions were stopped by chilling on ice and adding 15 µl of 0.5 M EDTA, pH 8.0. Reaction products were extracted with phenol-chloroform-isoamyl alcohol (25:24:1), and DNA fragments were further purified with CENTRISEP columns (Princeton Separations). For fragment separation, a 5-µl aliquot of each sample was loaded onto an ABI 3730XL DNA analyzer. Sample files with output data from each run were automatically created by ABI 3730XL and analyzed with GeneMapper Analysis software.

### EMSAs.

EMSAs were performed with LightShift chemiluminescent EMSA kits (Life Technologies) according to the manufacturer’s instructions**.** Briefly, the biotinylated PCR product containing the *pmt* promoter region (obtained through PCR with MW2 genomic DNA and primers PpmtR-for and PpmtR-BTN-rev; 20 fM) or a random DNA fragment at the same concentration was incubated with 146 nM purified GST-PmtR fusion protein in a 20-µl reaction mixture for 20 min at room temperature. Whenever indicated, synthetic PSMs were used at a final concentration of 212 nM. The reaction mixtures were run on a 6% nondenaturing acrylamide gel, transferred onto NY+ membrane, and developed in accordance with the manufacturer’s recommendations. For antibody detection of the PmtR complex bound to DNA, the gel was transferred to nitrocellulose and analyzed by immunodetection with PmtR-specific antiserum (1:1,000) that was developed in rabbits against GST-PmtR fusion protein. After blocking and washing, the gel was incubated with an anti-rabbit IgG-Cy5 conjugate (1:5,000) and reaction products were visualized by using fluorescence at 700 nm on an Odyssey infrared imager (LiCor). An increased Ppmt* concentration (13 pM) was used to detect a sufficient amount of protein, as suggested by Khoury Christianson and Kafatos (33).

### Bioinformatic and statistical analyses.

Statistical analyses were performed with GraphPad Prism 6. Direct comparisons of two groups were analyzed with two-tailed *t* tests and multiple comparisons were analyzed by analysis of variance (ANOVA) with Tukey’s posttest. All error bars represent standard deviations (SDs). Protein sequence analysis was performed with BLASTP or ClustalW.

### Accession number(s).

Microarray results were deposited in GEO (http://www.ncbi.nlm.nih.gov/geo/) under accession number GSE72878.

## SUPPLEMENTAL MATERIAL

Figure S1  EMSA of the specificity of PmtR-P*pmt* and PSM-DNA interaction. (A) Specificity of interaction of PmtR with DNA. PmtR binds to P*pmt* but not to a random DNA fragment. (B) PSMα peptides bind nonspecifically to DNA. PSMα1, PSMα2, and PSMα4 show binding to a random DNA fragment. Download Figure S1, TIF file, 1.3 MB

Figure S2  Immunoblot analysis of EMSA. The EMSA of PmtR-DNA interaction with PSMα1 and PSMα2 was performed as for Fig. 5B, and the blot was probed with anti-PmtR antiserum. Black arrow, presumable PmtR monomer; white arrows, presumable PmtR aggregates; white circle, area where PSM-DNA complexes run in the EMSA shown in Fig. 5B. Note absence of PmtR reaction. Download Figure S2, TIF file, 2.5 MB

Figure S3  PSM production in and growth of MW2 wild-type and *pmtR* deletion strains. (A) PSM production measured by RP-HPLC/ESI-MS after growth in TSB for 24 h. (B) Growth in M9 synthetic medium. Experiments were performed in triplicate. Error bars show SDs. Download Figure S3, TIF file, 2.5 MB

Table S1  Microarray analysis of genes differentially expressed in strain MW2 versus PSM-deficient isogenic MW2 Δ*psmαβhld* Table S1, PDF file, 0.1 MB

Table S2  Strains, plasmids, and primers used in this study. Table S2, PDF file, 0.1 MB
