# Supplementary figures and images for: Mechanism of Gene Regulation by a Staphylococcus aureus Toxin
Source: mBio. 2016 Oct 25;7(5):e01579-16. doi: 10.1128/mBio.01579-16 (PMC5080381; doi:10.1128/mBio.01579-16)

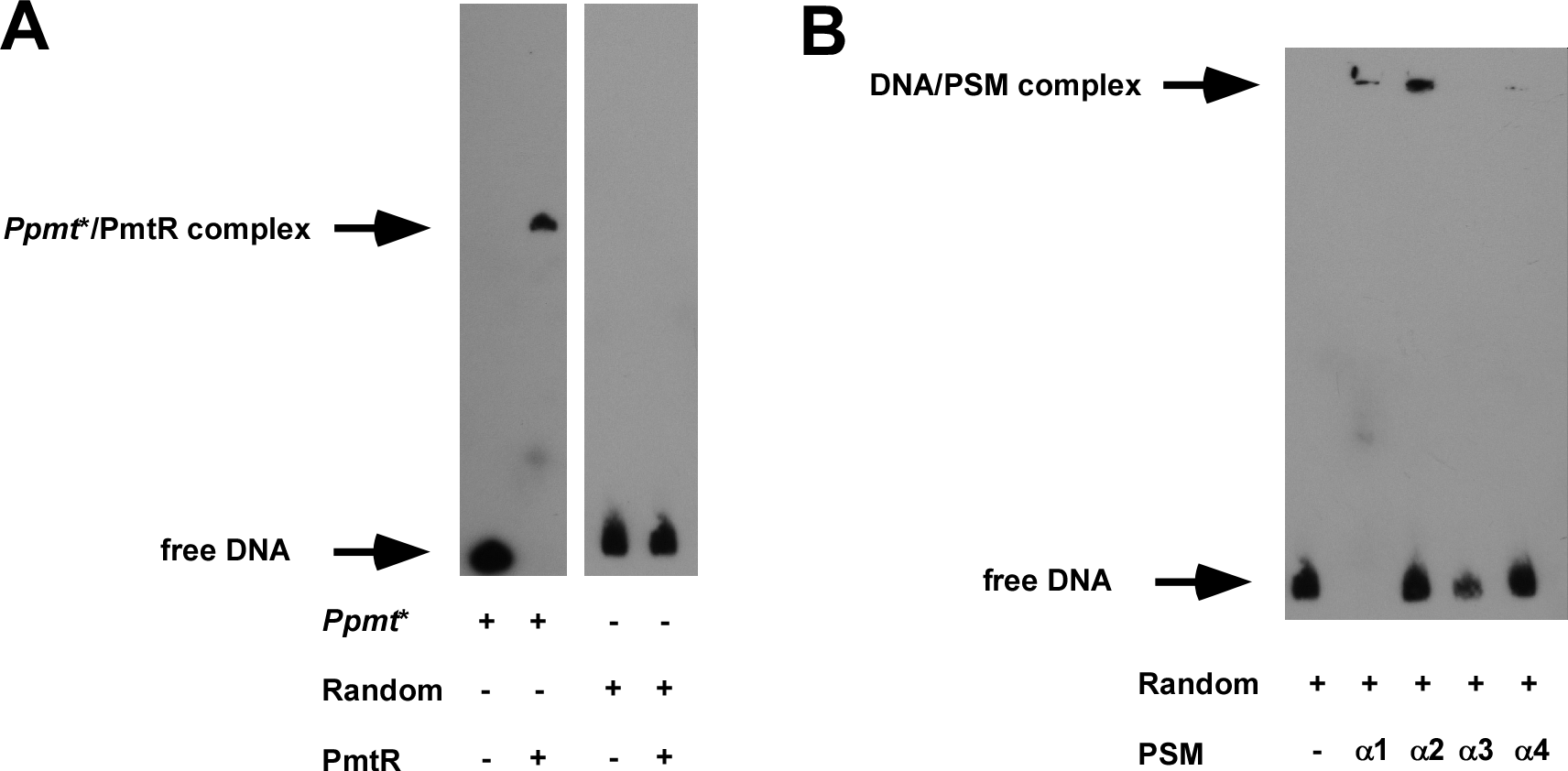

Supplement: Figure S1 — EMSA of the specificity of PmtR-Ppmt and PSM-DNA interaction. (A) Specificity of interaction of PmtR with DNA. PmtR binds to Ppmt but not to a random DNA fragment. (B) PSMα peptides bind nonspecifically to DNA. PSMα1, PSMα2, and PSMα4 show binding to a random DNA fragment. Download [file mbo005163039sf1.tif]

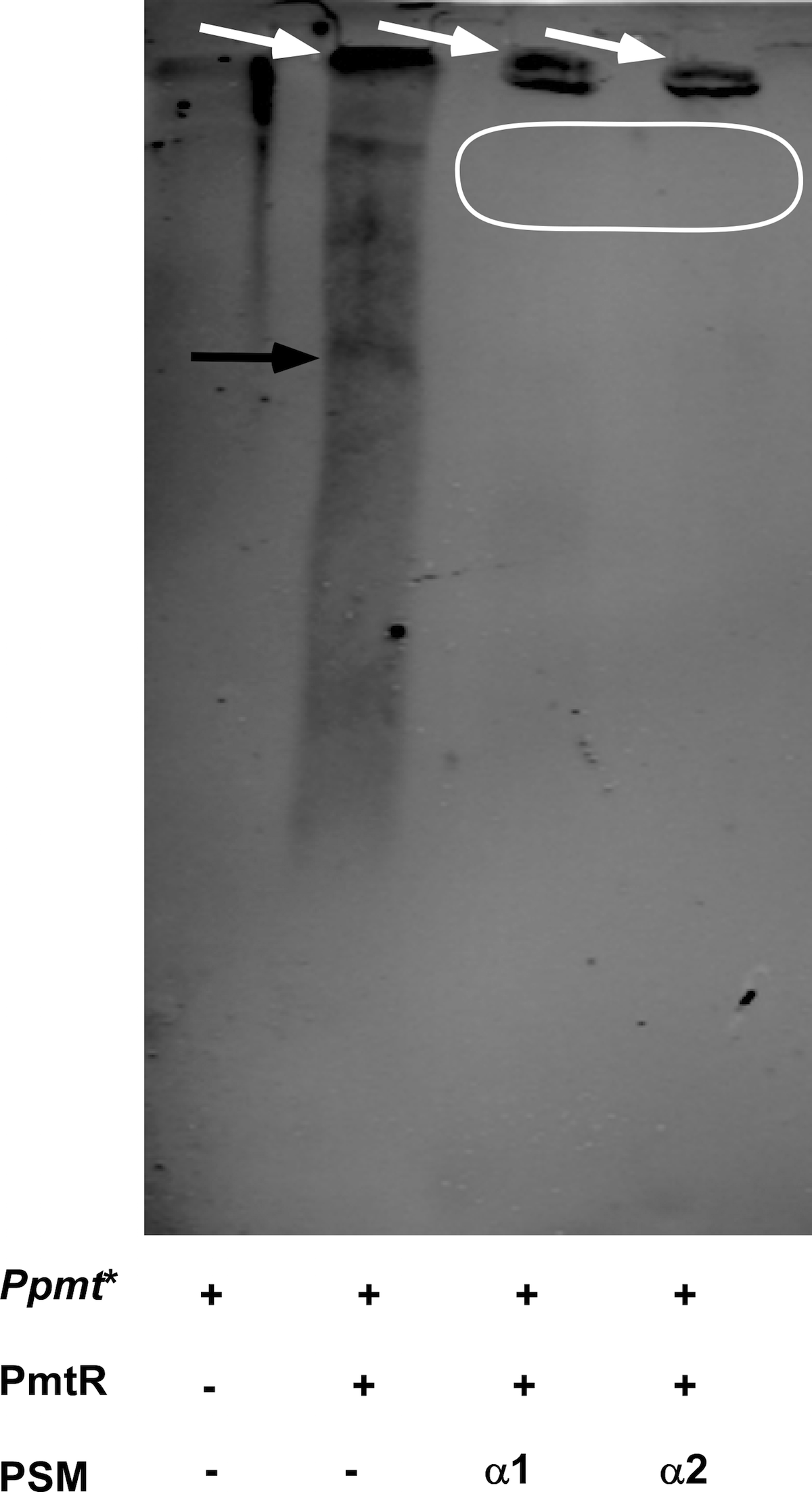

Supplement: Figure S2 — Immunoblot analysis of EMSA. The EMSA of PmtR-DNA interaction with PSMα1 and PSMα2 was performed as for Fig. 5B, and the blot was probed with anti-PmtR antiserum. Black arrow, presumable PmtR monomer; white arrows, presumable PmtR aggregates; white circle, area where PSM-DNA complexes run in the EMSA shown in Fig. 5B. Note absence of PmtR reaction. Download [file mbo005163039sf2.tif]

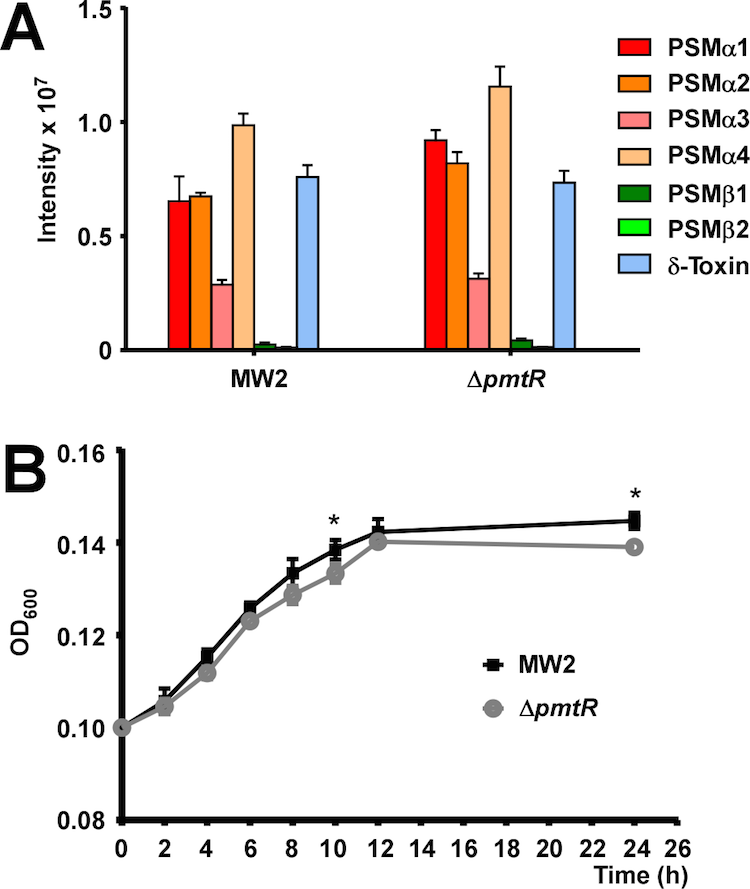

Supplement: Figure S3 — PSM production in and growth of MW2 wild-type and pmtR deletion strains. (A) PSM production measured by RP-HPLC/ESI-MS after growth in TSB for 24 h. (B) Growth in M9 synthetic medium. Experiments were performed in triplicate. Error bars show SDs. Download [file mbo005163039sf3.tif]
