# Supplementary material for: Mechanism of Gene Regulation by a Staphylococcus aureus Toxin
Source: mBio. 2016 Oct 25;7(5):e01579-16. doi: 10.1128/mBio.01579-16 (PMC5080381; doi:10.1128/mBio.01579-16)
Supplement: Table S1 — Microarray analysis of genes differentially expressed in strain MW2 versus PSM-deficient isogenic MW2 Δpsmαβhld [file mbo005163039st1.pdf]

Table S1. Microarray analysis of genes differentially expressed in strain MW2 versus the PSM-deficient isogenic MW2Δ*psm*αβ*hld*[1].

Gene number	Gene function	Gene name	Factor of regulation
	Up-regulated in MW2 versus MW2Δ*psm*αβ*hld*		
MW2207	urease subunit beta	*ureB*	9.5
MW0528	hypothetical protein	*proP*	9.5
MW0135	capsular polysaccharide synthesis enzyme Cap8L	*cap8L*	9.4
MW0137	capsular polysaccharide synthesis enzyme Cap8N	*cap8N*	9.2
MW0133	capsular polysaccharide synthesis enzyme Cap8J	*cap8J*	9.2
MW2208	urease subunit alpha	*ureC*	9.1
MW2575	hypothetical protein	-	8.8
MW2206	urease subunit gamma	*ureA*	8.5
MW0134	capsular polysaccharide synthesis enzyme Cap8K	*cap8K*	7.9
MW2212	urease accessory protein UreD	*ureD*	7.8
MW0131	capsular polysaccharide synthesis enzyme Cap8H	*cap8H*	7.7
MW0129	capsular polysaccharide synthesis enzyme Cap8F	*cap8F*	7.3
MW2600	hypothetical protein	-	7.3
MW0132	capsular polysaccharide synthesis enzyme Cap8I	*cap8I*	7.2
MW2209	urease accessory protein UreE	*ureE*	7.1
MW0259	hypothetical protein	-	7.1
MW0256	hypothetical protein	-	6.9
MW0257	hypothetical protein	-	6.7
MW2210	urease accessory protein UreF	*ureF*	6.6
MW0518	Ser-Asp rich fibrinogen-binding bone sialoprotein-binding protein	*sdrE*	6.5
MW0263	hypothetical protein	-	6.4
MW2572	hypothetical protein	-	6.3
MW0258	hypothetical protein	-	6.2
MW2541	hypothetical protein	-	6.1
MW0136	capsular polysaccharide synthesis enzyme Cap8M	*cap8M*	6.0
MW0130	capsular polysaccharide synthesis enzyme Cap8G	*cap8G*	5.9
MW2505	hypothetical protein	-	5.9
MW0265	hypothetical protein	-	5.8
MW2444	D-lactate dehydrogenase	*ddh*	5.7
MW0128	capsular polysaccharide synthesis enzyme Cap8E	*cap8E*	5.7
MW2205	hypothetical protein	-	5.4
MW2505	hypothetical protein	-	5.2
MW0266	hypothetical protein	-	5.2
MW0081	myosin-cross-reactive antigen	-	5.2
MW0267	hypothetical protein	-	5.0
MW2574	preprotein translocase subunit SecY	*secY*	5.0
	Down-regulated in MW2 versus MW2Δ*psm*αβ*hld*		
MW0767	extracellular ECM and plasma binding protein	*ssp*	-8.5
MW0534	hypothetical protein	-	-5.3

[1] All genes with a factor of regulation ≥ 5.0 are shown. See GEO accession number GSE72878 for the entire list.
