# Supplementary material for: Mechanism of Gene Regulation by a Staphylococcus aureus Toxin
Source: mBio. 2016 Oct 25;7(5):e01579-16. doi: 10.1128/mBio.01579-16 (PMC5080381; doi:10.1128/mBio.01579-16)
Supplement: Table S2 — Strains, plasmids, and primers used in this study. [file mbo005163039st2.pdf]

Table S2. Strains, plasmids, and primers used in the present study.

	Designation/sequence	Reference/source/purpose
**Strains**		
MW2 (WT)	*S. aureus* MW2, community-associated MRSA strain	(Baba et al., 2002)[1]
Δ*psmαβhld*	*S. aureus* MW2 with *psm*α and *psm*β operons deleted and the *hld* start codon altered to abolish translation	(Cheung et al., 2012)
Δ*psmαβhld*Δ*pmtR*	Δ*psmαβhld* with *pmtR* gene deleted	This study
Δ*agr*	*S. aureus* MW2 with *agr* system deleted	(Wang et al., 2007)
E. coli BL21 (DE3)	*E. coli* host for protein expression<br>*fhuA2 [lon] ompT gal (λ DE3) [dcm]* Δ*hsdS*<br>*λ DE3 = λ sBamHIo* Δ*EcoRI-B*<br>*int::(lacI::PlacUV5::T7 gene1) i21* Δ*nin5*	Life Technologies
**Plasmids**		
pTX$_{\Delta}$*pmtR*	Constitutive expression vector expressing PmtR; tet^R	This study
pTX$_{\Delta}$*16*	Control vector for pTX$_{\Delta}$ plasmid series; tet^R	(Wang et al., 2007)
pTXpsmα	Xylose-inducible expression vector expressing PSMα1-4; tet^R	(Chatterjee et al., 2013)
pKX16	Control vector for pKX plasmid series; kan^R	(Chatterjee et al., 2011)
pKXpsmα	pKX expressing PSMα1-4 upon xylose induction; kan^R	This study
pKXpsmβ	pKX expressing PSMβ1-2 upon xylose induction; kan^R	This study
pKXhld	pKX expressing δ-toxin upon xylose induction; kan^R	This study
pGEX-4T-1	Vector for GST fusion protein cloning and expression; amp^R	GE Healthcare
pGEX-4T-1gst-pmtR	Vector expressing GST-PmtR; amp^R	This study
pTX16	Control vector for pTX plasmid series; tet^R	(Peschel et al., 1996)
pTXpsmα1	pTX expressing PSMα1 upon xylose induction; tet^R	This study
pTXpsmα2	pTX expressing PSMα2 upon xylose induction; tet^R	This study
pTXpsmα3	pTX expressing PSMα3 upon xylose induction; tet^R	This study
pTXpsmα4	pTX expressing PSMα4 upon xylose induction; tet^R	This study
**Primers**		
pmtR-P1	ggggacaagtttgtacaaaaaagcaggctttccgtctttgtttgtcgcc atacctatagttgc	Deletion of *pmtR*
pmtR-P2	caacgtccccctatcactttctg	Deletion of *pmtR*
pmtR-P3	gtgataggggggacgttgatgaatgccatagaattaagtaatg	Deletion of *pmtR*
pmtR-P4	ggggaccactttgtacaagaaagctgggttaggtaaaagaactttgtt gagtcaagc	Deletion of *pmtR*
pmtR-for	gaggatcctagggggacgttgatgaaaataattttaaaaaacaatag	Cloning of *pmtR* into pTX$_{\Delta}$
pmtR-rev	tctaacgcgttcatgatgattcctcctcataaatgaacg	Cloning of *pmtR* into pTX$_{\Delta}$
GST-pmtR-for	gtggaggatccgagaacctgtacttccagggtatgaaaataattttaa aaaacaatagtgattttccgatttatgaacagattaagc	Cloning of *pmtR* into pGEX-4T-1

GST-pmtR-rev	cagtcgactcatgatgattcctcctcataaatgaacg	Cloning of *pmtR* into pGEX-4T-1
PpmtR-for	agacattcgttttaggtacctttttattg	Amplification of *pmtR* promoter
PpmtR-rev	caacgtccccctatcactttctgtatatatac	Amplification of *pmtR* promoter
PpmtR-for[BTN]	[BTN]caacgtccccctatcactttctgtatatatacaatatacac	Amplification of biotin-labeled *pmtR* promoter
PpmtRmut1-rev	caacgtccccctatcactttctgtatatatacaatatacacaatvtacac agttttg	Introduction of 1-bp mutation in *pmtR* promoter
PpmtRmut1-for	caacgtccccctatcactttccgtatatatacaatatacacaatatacac agttttg	Introduction of 1-bp mutation in *pmtR* promoter
M13L	gtaaaacgacggccag	Fragment analysis (Queck et al., 2008)
M13R	caggaaacagctatgac	Fragment analysis (Queck et al., 2008)
*M13L	[FAM]gtaaaacgacggccag	Fragment analysis (Queck et al., 2008)
*M13R	[FAM]caggaaacagctatgac	Fragment analysis (Queck et al., 2008)

5'RACE

Pmtrace1	aattctttaaccaaattttcaatg	RNA adapter, PCR
Pmtrace2	taccagcgccatttctacc	PCR
Pmtraceseq	ctcttttaaaatagaactatcttg	Sequencing

Primers and probes for qRT-PCR

pmt-for	cgtagagtcaaagtgccatatggt	qRT-PCR of *pmtB*
pmt-probe	caattgcaatagtgttggtgaa	qRT-PCR of *pmtB*
pmt-rev	tgggaatgatgattgacttagaagaa	qRT-PCR of *pmtB*

Randomized DNA fragment for EMSA control

AATCACAATCTACTACCTTACTGGAATTTAATTTATCTTATAATTTCCCTTGCATATAAGTTACAT
TACTTCAGCCTCCTAATTGTACCCTTAGCACGAAGACAAATTTGTTCTTACCTATATTTCATCATT
GGTAAGGGGATTGCATGTCCCACGTAAAACATTGTTAAACTCTTAGGTTTTTGAACGATAAAAAC
TTT

[1] References

Baba, T., Takeuchi, F., Kuroda, M., Yuzawa, H., Aoki, K., Oguchi, A., Nagai, Y., Iwama, N., Asano, K., Naimi, T., *et al.* (2002). Genome and virulence determinants of high virulence community-acquired MRSA. Lancet *359*, 1819-1827.

Chatterjee, S.S., Chen, L., Joo, H.S., Cheung, G.Y., Kreiswirth, B.N., and Otto, M. (2011). Distribution and regulation of the mobile genetic element-encoded phenol-soluble modulin PSM-mec in methicillin-resistant *Staphylococcus aureus*. PLoS One *6*, e28781.

Chatterjee, S.S., Joo, H.S., Duong, A.C., Dieringer, T.D., Tan, V.Y., Song, Y., Fischer, E.R., Cheung, G.Y.C., Li, M., and Otto, M. (2013). Essential *Staphylococcus aureus* toxin export system. Nat Med *19*, 364-367.

Cheung, G.Y., Duong, A.C., and Otto, M. (2012). Direct and synergistic hemolysis caused by *Staphylococcus* phenol-soluble modulins: implications for diagnosis and pathogenesis. Microbes Infect *14*, 380-386.

Peschel, A., Ottenwalder, B., and Gotz, F. (1996). Inducible production and cellular location of the epidermin biosynthetic enzyme EpiB using an improved staphylococcal expression system. FEMS Microbiol Lett *137*, 279-284.

Queck, S.Y., Jameson-Lee, M., Villaruz, A.E., Bach, T.H., Khan, B.A., Sturdevant, D.E., Ricklefs, S.M., Li, M., and Otto, M. (2008). RNAIII-independent target gene control by the agr quorum-sensing system: insight into the evolution of virulence regulation in *Staphylococcus aureus*. Mol Cell *32*, 150-158.
Wang, R., Braughton, K.R., Kretschmer, D., Bach, T.H., Queck, S.Y., Li, M., Kennedy, A.D., Dorward, D.W., Klebanoff, S.J., Peschel, A.*, et al.* (2007). Identification of novel cytolytic peptides as key virulence determinants for community-associated MRSA. Nat Med *13*, 1510-1514.
